# Effects of dietary acidifiers and other low acid-binding capacity formulation strategies on weanling pig growth performance and fecal dry matter

**DOI:** 10.1093/tas/txaf104

**Published:** 2025-08-19

**Authors:** Ethan B Stas, Mike D Tokach, Jason C Woodworth, Joel M DeRouchey, Robert D Goodband, Jordan T Gebhardt

**Affiliations:** Department of Animal Sciences and Industry, Kansas State University, Manhattan KS 66506-0201USA; Department of Animal Sciences and Industry, Kansas State University, Manhattan KS 66506-0201USA; Department of Animal Sciences and Industry, Kansas State University, Manhattan KS 66506-0201USA; Department of Animal Sciences and Industry, Kansas State University, Manhattan KS 66506-0201USA; Department of Animal Sciences and Industry, Kansas State University, Manhattan KS 66506-0201USA; Department of Diagnostic Medicine/Pathobiology, College of Veterinary Medicine, Kansas State University, Manhattan KS, 66506-0201USA

**Keywords:** Acid-binding capacity, acidifiers, calcium, fecal dry matter, lactose, weanling pigs

## Abstract

Two experiments were conducted to determine effects of dietary acidifiers and low acid-binding capacity at a pH of 4 (ABC-4) formulation strategies on weanling pig performance and fecal dry matter (DM). In Exp. 1, 300 pigs, initially 6.1 kg, were allotted to 1 of 6 dietary treatments fed in two phases with 5 pigs per pen and 10 replications per treatment. Treatment diets were formulated using four acidifiers to target an ABC-4 of 200 and 250 meq/kg in phases 1 and 2, respectively. The acidifiers included fumaric acid (Primary Products Ingredients Americas LLC, Decatur, IL), Activate DA (Novus, St. Charles, MO), KEM-GEST (Kemin, Des Moines, IA), and ACID-AID (Alltech, Nicholasville, KY) added at 0.36, 0.87, 1.01, and 0.84% of the diet, respectively. The fifth treatment did not contain acidifiers which increased the ABC-4 value of the diet by 40 meq/kg. The last treatment was the same formulation as the diet without acidifiers, but with the addition of pharmacological levels of Zn from ZnO. From d 0 to 24 and d 0 to 38, pigs fed low ABC-4 diets had increased (*P *≤ 0.020) G:F compared to pigs fed the high ABC-4 diet. In Exp. 2, 725 pigs, initially 5.9 kg, were allotted to 1 of 5 dietary treatments fed in two phases with 5 pigs per pen and 29 replications per treatment. Three treatments were formulated using 0.46% fumaric acid with an additional low ABC-4 formulation strategy to target the same low ABC-4 level as Exp. 1. The three strategies consisted of low Ca levels, 0.6% formic acid (Amasil NA; BASF; Florham Park, NJ), or replacing whey permeate with crystalline lactose. The fourth treatment was formulated to be 100 meq/kg greater than the low ABC-4 diets. The final treatment was the same formulation as the high ABC-4 diet but with the addition of pharmacological levels of Zn. From d 0 to 24 and d 0 to 38, pigs fed the crystalline lactose diet had decreased (*P *< 0.05) ADFI compared to the other low ABC-4 formulation strategies. Pigs fed low ABC-4 diets had increased (*P *≤ 0.024) G:F and fecal DM compared to the 100 meq/kg higher ABC-4 diet. From d 0 to 24, pigs fed the diet containing ZnO had increased (*P *≤ 0.001) ADG and ADFI compared to the high ABC-4 diet without ZnO. In summary, the combination of fumaric and formic acid had the best performance among low ABC-4 formulation strategies, with pigs fed low ABC-4 diets having improved feed efficiency and fecal DM compared to a higher ABC-4 diet when the diets did not contain ZnO.

## Teaser

Various dietary acidifiers and low ABC-4 formulation strategies can be used to improve performance and fecal dry matter of newly weaned pigs in diets without ZnO.

## INTRODUCTION

Dietary acidifiers are commonly used in weanling pig diets as a method to reduce pH in the gastrointestinal tract promoting nutrient digestibility and inhibiting pathogenic bacteria (Pluske et al., 2021). However, dietary acidifiers are often added without considering the effect other ingredients have on the pH of the gastrointestinal tract. Low acid-binding capacity formulated diets consider the impact of ingredient combinations in the diet on weanling pig stomach pH opposed to the effect of an individual ingredient. Specifically, acid-binding capacity at a pH of 4 (ABC-4) is the amount of hydrochloric acid required to achieve a stable pH of 4 for 1 kg of ingredient or diet (Lawlor et al., 2005; Stas et al., 2022). Previous research has observed improvements in growth performance and fecal dry matter (DM) when diets are formulated to 200 meq/kg from d 0 to 10 post-weaning followed by 250 meq/kg from d 10 to 24 post-weaning ( Stas et al., 2025). The use of dietary acidifiers is a simple strategy that can be incorporated to lower the overall ABC-4 value of the complete diet without affecting other nutrients or ingredients (Stas et al., 2022). However, dietary acidifiers differ in their individual ingredient ABC-4 values because of different characteristics such as inorganic vs. organic origins, differences in pKa values, commercial products that are based on acid blends, or final product presentation as an acid salt. Therefore, different dietary acidifiers will likely have different effects on the stomach acidity of young pigs if they are incorporated at the same rate (Lawlor et al., 2005; Wang et al., 2023).

There has been extensive research on the use of dietary acidifiers and overall, it appears to be positively correlated with growth performance (Wang et al., 2022). However, there is little research evaluating acidifiers in combination with low ABC-4 formulation strategies. A review by Kil et al. (2011) reported that although dietary acidifiers aid in reducing the pH of the stomach in weanling pigs, the reduction in pH is often not large enough to improve nutrient digestion and prevent proliferation of pathogenic organisms. This observation may be due to other ingredients within the diet that are contributing to an elevated stomach pH and thus limiting dietary acidifier’s effectiveness. Using dietary acidifiers in combination with low ABC-4 formulation strategies may allow better acidification of the gastrointestinal tract to improve growth performance and lower the occurrence of post-weaning diarrhea. In addition to dietary acidifiers, there are various strategies that can be used to lower the ABC-4 level of the diet. Lowering the Ca level of the diet by reducing the amount of calcium carbonate has been observed to increase growth performance in weanling pigs (Wu et al., 2018; Warner et al., 2023). The use of specialty protein sources and lactose sources that have low ABC-4 values has been observed to improve feed efficiency in weanling pigs ( Zhao et al., 2021; Stas et al., 2025). Using multiple acidifiers with low ABC-4 values can have synergistic effects because of their different pKa values as well as their combined ability to further reduce the ABC-4 (Nguyen et al., 2020). Evaluation of dietary acidifiers and formulation strategies to target the same low ABC-4 level is warranted to determine if the response is altered in newly weaned pigs.

Therefore, the objective of these studies was to determine the effects of different dietary acidifiers and low ABC-4 formulation strategies on weanling pig performance and fecal DM. Our hypothesis was that different dietary acidifiers and low ABC-4 formulation strategies will elicit a similar response when used to target the same ABC-4 level of 200 meq/kg from d 0 to 10 post-weaning and 250 meq/kg from d 10 to 24 post-weaning resulting in improved weanling pig performance and fecal DM compared to higher ABC-4 levels.

## MATERIALS AND METHODS

### General

The Kansas State University Institutional Animal Care and Use Committee approved the protocols used in these experiments. Experiment 1 was conducted at the Kansas State University Swine Teaching and Research Center in Manhattan, KS. The study was conducted in two identical nursery rooms. Experiment 2 was conducted in two research facilities, the Kansas State University Swine Teaching and Research Center and the Kansas State University Segregated Early Weaning facility, both located in Manhattan, KS. The first facility used in Exp. 2 was a single nursery room and the second facility was two identical nursery barns. All facilities in both experiments were completely enclosed, environmentally controlled, and mechanically ventilated. Each pen contained a 4-hole, dry self-feeder and a nipple waterer to provide ad libitum access to feed and water. Pens (1.2 × 1.2 m) had tri-bar floors and allowed approximately 0.30 m^2^/pig.

#### ABC-4 analysis.

A sample of each ingredient and complete diet were analyzed for ABC-4 at the Kansas State University Swine Nutrition Laboratory in Manhattan, KS (Table 1). Analysis was accomplished by the procedure outlined by Stas et al. (2022). Each ingredient and diet were analyzed in triplicate and the average of each analysis was used for the overall analyzed ABC-4 value. Individual ingredient ABC-4 values were used for experimental diet formulation. Complete diets were analyzed to confirm dietary ABC-4 formulation from ingredient values.

**Table 1.** Analyzed acid-binding capacity at a pH of 4 (ABC-4) of ingredients used in experimental diets^1^

Ingredient, %	Initial pH	ABC-4 (meq/kg)
Corn	6.84	84
Soybean meal	7.04	602
Crystalline lactose	4.61	53
Whey permeate^2^	6.48	433
Microbially enhanced soybean meal^3^	5.11	67
Spray-dried bovine plasma	6.99	713
Fumaric acid^4^	2.31	-10,873
Formic acid^5^	3.02	-8,287
Activate DA^6^	2.99	-4,487
KEM-GEST^7^	2.06	-3,800
ACID-AID^8^	2.21	-4,680
Limestone	9.70	18,384
Monocalcium phosphate	4.22	73
Salt	6.06	15
L-Lys	5.81	83
DL-Met	5.39	137
L-Thr	5.51	160
L-Trp	5.12	120
L-Val	5.52	193
ZnO	9.15	21,863
Vitamin premix with phytase	6.04	3,210
Vitamin premix without phytase	7.07	10,767
Trace mineral premix	5.13	7,867
Phytase	5.95	170

^1^Acid-binding cpacity-4 was determined by the amount of 0.1 N HCl required to lower the initial pH of a sample to a stable pH of 4.00 ± 0.04. Acidifiers were titrated with 0.1 N NaOH to raise the initial pH to a stable pH of 4.00 ± 0.04. Each sample was analyzed in triplicate.

^2^Dairylac 80; International Ingredients Corporation; Fenton, MO.

^3^MEPRO; Prairie Aquatech; Brookings, SD.

^4^Primary Products Ingredients Americas LLC, Decatur, IL.

^5^Amasil NA, BASF, Florham Park, NJ.

^6^Novus; St. Charles, MO.

^7^Kemin; Des Moines, IA.

^8^Alltech; Nicholasville, KY.

### Experiment 1

#### Animals and treatment structure.

The objective of Exp. 1 was to determine the effects of various commercial dietary acidifiers formulated to an equal ABC-4 level on nursery pig performance and fecal DM. A total of 300 weanling pigs (DNA 241 × 600; initially 6.1 ± 0.02 kg BW) were used in a 38-d trial. At weaning, pens of pigs were randomly allotted to 1 of 6 dietary treatments with 10 replications per treatment (Tables 2 and 3). Dietary treatments were fed in two phases with phase 1 provided from d 0 to 10 post-weaning, followed by phase 2 until d 24 post-weaning. Dietary treatments consisted of four low ABC-4 diets formulated to 200 and 250 meq/kg in phase 1 and 2, respectively. The four low ABC-4 diets utilized four different dietary acidifiers to achieve the low ABC-4 targets. Dietary acidifiers consisted of fumaric acid (Primary Products Ingredients Americas LLC, Decatur, IL), Activate DA (Novus, St. Charles, MO), KEM-GEST (Kemin, Des Moines, IA), and ACID-AID (Alltech, Nicholasville, KY) included at 0.36, 0.87, 1.01, and 0.84% of the diet in both phases, respectively. Fumaric acid, Activate DA, KEM-GEST, and ACID-AID had individual ingredient ABC-4 values of -10,873, -4,487, -3,800, and -4,680 meq/kg, respectively which ultimately determined their inclusion to achieve the target ABC-4 of experimental diets. The level of Activate DA, KEM-GEST, and ACID-AID in the diets exceeded supplier-recommended inclusion rates to achieve the low ABC-4 levels of 200 and 250 meq/kg in phase 1 and 2, respectively. The fifth treatment did not contain any dietary acidifiers which increased the ABC-4 level by 40 meq/kg in both phases. All five dietary treatments contained 100 mg/kg of Zn from Zn sulfate provided by the trace mineral premix and did not contain any pharmacological levels of Zn. The sixth and final dietary treatment was the same formulation as the fifth diet containing no acidifiers, but with the addition of 3,000 and 2,000 mg/kg of Zn from ZnO in phases 1 and 2, respectively. The addition of ZnO increased the diet ABC-4 value by 87 and 54 meq/kg in phases 1 and 2, respectively. The basal diets for phases 1 and 2 were manufactured at Hubbard feeds in Beloit, KS. The basal diets were divided into 6 batches and treatment ingredients were added and mixed at the Kansas State University O.H. Kruse Feed and Technology Innovation Center in Manhattan, KS to form the six experimental diets.

**Table 2.** Phase 1 diet composition (as-fed basis), Exp. 1^1^

	Dietary acidifier					
	1	2	3	4	-	-
ZnO:	-	-	-	-	-	+
ABC-4, meq/kg:	200	200	200	200	240	327
Ingredients						
Corn	54.65	54.14	54.00	54.18	55.01	54.61
Soybean meal	12.00	12.00	12.00	12.00	12.00	12.00
Crystalline lactose	15.00	15.00	15.00	15.00	15.00	15.00
Microbially enhanced soybean meal^2^	10.00	10.00	10.00	10.00	10.00	10.00
Spray-dried bovine plasma	2.50	2.50	2.50	2.50	2.50	2.50
Acidifier 1^3^	0.36	---	---	---	---	---
Acidifier 2^4^	---	0.87	---	---	---	---
Acidifier 3^5^	---	---	1.01	---	---	---
Acidifier 4^6^	---	---	---	0.84	---	---
ZnO	---	---	---	---	---	0.40
Corn oil	2.00	2.00	2.00	2.00	2.00	2.00
Limestone	0.26	0.26	0.26	0.26	0.26	0.26
Monocalcium phosphate	0.98	0.98	0.98	0.98	0.98	0.98
Salt	0.83	0.83	0.83	0.83	0.83	0.83
L-Lys	0.46	0.46	0.46	0.46	0.46	0.46
DL-Met	0.22	0.22	0.22	0.22	0.22	0.22
L-Thr	0.19	0.19	0.19	0.19	0.19	0.19
L-Trp	0.07	0.07	0.07	0.07	0.07	0.07
L-Val	0.10	0.10	0.10	0.10	0.10	0.10
Vitamin premix with phytase^7^	0.25	0.25	0.25	0.25	0.25	0.25
Trace mineral premix^8^	0.15	0.15	0.15	0.15	0.15	0.15
Total	100	100	100	100	100	100
Calculated analysis						
SID amino acids, %						
Lys	1.35	1.35	1.35	1.35	1.35	1.35
Ile:Lys	55	55	55	55	55	55
Leu:Lys	119	118	118	118	119	119
Met:Lys	35	35	35	35	35	35
Met and Cys:Lys	56	56	56	56	56	56
Thr:Lys	63	63	63	63	63	63
Trp:Lys	20.3	20.3	20.3	20.3	20.3	20.3
Val:Lys	70	70	70	70	70	70
His:Lys	36	36	36	36	36	36
Total Lys, %	1.51	1.51	1.51	1.51	1.51	1.51
NE, kcal/kg	2,619	2,606	2,604	2,608	2,630	2,619
SID Lys:NE, g/Mcal	5.15	5.18	5.18	5.18	5.14	5.16
CP, %	20.3	20.3	20.3	20.3	20.4	20.3
Ca, %	0.46	0.46	0.46	0.46	0.46	0.46
P, %	0.51	0.51	0.51	0.51	0.51	0.51
STTD P, %	0.46	0.46	0.46	0.46	0.46	0.46
Analyzed ABC-4, meq/kg	173	173	173	173	213	267

^1^Phase 1 diets were fed from d 0 to 10 post-weaning.

^2^ME-PRO; Prairie Aquatech; Brookings, SD.

^3^Fumaric acid; Primary Products Ingredients Americas LLC, Decatur, IL.

^4^Activate DA; Novus; St. Charles, MO.

^5^KEM-GEST; Kemin; Des Moines, IA.

^6^ACID-AID; Alltech; Nicholasville, KY.

^7^Provided per kg of diet: 4,134 IU vitamin A; 1,653 IU vitamin D; 44 IU vitamin E; 3 mg vitamin K; 0.03 mg vitamin B12; 50 mg niacin; 28 mg pantothenic acid; 8 mg riboflavin. Quantum Blue 5G (ABVista, Plantation, FL) provided an estimated release of 0.13% STTD P with 1,250 FTU/kg.

^8^Provided per kg of diet: 110 mg Zn from zinc sulfate; 110 mg Fe from iron sulfate; 33 mg Mn from manganese oxide; 17 mg Cu from copper sulfate; 0.30 mg I from calcium iodate; 0.30 mg Se from sodium selenite.

**Table 3.** Phase 2 diet composition (as-fed basis), Exp. 1^1^

	Dietary acidifier					
	1	2	3	4	-	-
ZnO:	-	-	-	-	-	+
ABC-4, meq/kg:	250	250	250	250	290	344
Ingredients, %						
Corn	60.60	60.09	59.94	60.12	60.96	60.71
Soybean meal	18.75	18.75	18.75	18.75	18.75	18.75
Crystalline lactose	7.50	7.50	7.50	7.50	7.50	7.50
Microbially enhanced soybean meal^2^	8.00	8.00	8.00	8.00	8.00	8.00
Acidifier 1^3^	0.36	---	---	---	---	---
Acidifier 2^4^	---	0.87	---	---	---	---
Acidifier 3^5^	---	---	1.01	---	---	---
Acidifier 4^6^	---	---	---	0.84	---	---
ZnO	---	---	---	---	---	0.25
Corn oil	1.00	1.00	1.00	1.00	1.00	1.00
Limestone	0.41	0.41	0.41	0.41	0.41	0.41
Monocalcium phosphate	1.03	1.03	1.03	1.03	1.03	1.03
Salt	0.80	0.80	0.80	0.80	0.80	0.80
L-Lys	0.52	0.52	0.52	0.52	0.52	0.52
DL-Met	0.23	0.23	0.23	0.23	0.23	0.23
L-Thr	0.22	0.22	0.22	0.22	0.22	0.22
L-Trp	0.08	0.08	0.08	0.08	0.08	0.08
L-Val	0.12	0.12	0.12	0.12	0.12	0.12
Vitamin premix with phytase^7^	0.25	0.25	0.25	0.25	0.25	0.25
Trace mineral premix^8^	0.15	0.15	0.15	0.15	0.15	0.15
Total	100	100	100	100	100	100
Calculated analysis						
SID amino acids, %						
Lys	1.35	1.35	1.35	1.35	1.35	1.35
Ile:Lys	56	56	56	56	56	56
Leu:Lys	117	117	117	117	117	117
Met:Lys	37	37	37	37	37	37
Met and Cys:Lys	56	56	56	56	56	56
Thr:Lys	63	63	63	63	63	63
Trp:Lys	21.0	21.0	21.0	21.0	21.0	21.0
Val:Lys	70	70	70	70	70	70
His:Lys	36	36	36	36	36	36
Total Lys, %	1.50	1.50	1.50	1.50	1.50	1.50
NE, kcal/kg	2,524	2,511	2,507	2,511	2,533	2,526
SID Lys:NE, g/Mcal	5.35	5.38	5.39	5.38	5.34	5.35
CP, %	20.7	20.7	20.7	20.7	20.7	20.7
Ca, %	0.54	0.54	0.54	0.54	0.54	0.54
P, %	0.54	0.54	0.54	0.54	0.54	0.54
STTD P, %	0.46	0.46	0.46	0.46	0.46	0.46
Analyzed ABC-4, meq/kg	233	227	227	227	280	313

^1^Phase 2 diets were fed from d 10 to 24 post-weaning.

^2^ME-PRO; Prairie Aquatech; Brookings, SD.

^3^Fumaric acid; Primary Products Ingredients Americas LLC, Decatur, IL.

^4^Activate DA; Novus; St. Charles, MO.

^5^KEM-GEST; Kemin; Des Moines, IA.

^6^ACID-AID; Alltech; Nicholasville, KY.

^7^Provided per kg of diet: 4,134 IU vitamin A; 1,653 IU vitamin D; 44 IU vitamin E; 3 mg vitamin K; 0.03 mg vitamin B12; 50 mg niacin; 28 mg pantothenic acid; 8 mg riboflavin. Quantum Blue 5G (ABVista, Plantation, FL) provided an estimated release of 0.13% STTD P with 1,250 FTU/kg.

^8^Provided per kg of diet: 110 mg Zn from zinc sulfate; 110 mg Fe from iron sulfate; 33 mg Mn from manganese oxide; 17 mg Cu from copper sulfate; 0.30 mg I from calcium iodate; 0.30 mg Se from sodium selenite.

Individual pig weights and feed disappearance were measured on d 10 and weekly afterwards to determine ADG, ADFI, and G:F. Fecal samples were collected on d 10, 17, and 24 to determine percentage fecal DM from the same three medium weight pigs from each pen. After collection, fecal samples were dried at 55 °C in a forced air oven for 48 h and the ratio of dried to wet fecal weight determined percentage fecal DM.

### Experiment 2

#### Animals and treatment structure.

The objective of Exp. 2 was to determine the effect of dietary acidifiers and other low ABC-4 formulation strategies on weanling pig performance and fecal DM. A total of 725 pigs (DNA 200 × 400; DNA 241 × 600; initially 5.9 ± 0.09 kg BW) were used in a 38-d trial across two research facilities. At weaning, pens of pigs were randomly allotted to 1 of 5 dietary treatments with 29 replications per treatment. Dietary treatments were fed in two phases with phase 1 fed according to a feed budget of 2.3 kg/pig, followed by phase 2 diets which were fed until d 24 post-weaning (Tables 4 and 5). Dietary treatments consisted of three low ABC-4 diets formulated to 200 and 250 meq/kg in phases 1 and 2, respectively. The three low ABC-4 diets used 0.46% fumaric acid in combination with three other strategies to achieve the low ABC-4 level. The first strategy to lower the ABC-4 of the diet consisted of low Ca levels by decreasing the amount of limestone in the diet. Limestone has an individual ingredient ABC-4 value of 18,384 meq/kg. The low Ca treatment was formulated to have a Ca:P ratio 0.20 lower than all other dietary treatments. The second strategy utilized 0.6% formic acid (Amasil NA, BASF, Florham Park, NJ) in combination with the fumaric acid. The third strategy to lower ABC-4 consisted of replacing whey permeate (Dairylac 80, International Ingredients Corporation, Fenton, MO) with crystalline lactose based on lactose equivalency. Whey permeate and crystalline lactose have individual ingredient ABC-4 values of 433 and 53 meq/kg, respectively. This strategy led to an ABC-4 of 193 and 262 meq/kg in phases 1 and 2, respectively. Ultimately, a complete replacement of whey permeate with crystalline lactose resulted in an ABC-4 diet slightly below target for phase 1 and slightly above target for phase 2. However, the average ABC-4 value across the two phases is similar to the other low ABC-4 formulation strategies. The alternative lactose strategy adjusted the inclusion of microbially enhanced soybean meal (MEPRO; Prairie Aquatech; Brooking, SD) to maintain the same level of soybean meal across all diets. Salt inclusion was also adjusted with the inclusion of crystalline lactose to maintain a sodium level of 0.40 and 0.35% in phases 1 and 2, respectively. The fourth dietary treatment was formulated to an ABC-4 level of 300 and 350 meq/kg in phases 1 and 2, respectively. This diet did not contain any acidifiers, utilized only whey permeate as the lactose source, and had a Ca:P ratio of 0.9:1 and 1.0:1 in phases 1 and 2, respectively. All four dietary treatments contained 110 mg/kg of Zn from Zn sulfate provided by the trace mineral premix. The fifth and final dietary treatment was the same as the fourth diet, but with the addition of 3,000 and 2,000 mg/kg of Zn from ZnO in phases 1 and 2, respectively. The addition of ZnO increased the ABC-4 value by 87 and 54 meq/kg in phases 1 and 2, respectively. Diets were manufactured at the Kansas State University O.H. Kruse Feed and Technology Innovation Center in Manhattan, KS. Individual pig weights and feed disappearance were measured on d 8, 17, 24, 31, and 38 to determine ADG, ADFI, and G:F. Fecal samples were collected on d 8, 17, and 24 to determine fecal DM percentage and followed the same procedure outlined for Exp. 1.

**Table 4.** Phase 1 diet composition (as-fed basis), Exp. 2^1^

Fumaric acid:	+	+	+	-	-
Low ABC-4 Strategy:	Low Ca	Formic acid	Crystalline lactose	-	-
ZnO:	-	-	-	-	+
ABC-4, meq/kg:	200	200	193	300	387
Ingredients, %					
Corn	57.65	56.78	59.21	57.84	57.44
Soybean meal	12.00	12.00	12.00	12.00	12.00
Whey permeate^2^	15.00	15.00	---	15.00	15.00
Crystalline lactose	---	---	12.00	---	---
Microbially enhanced soybean meal^3^	7.50	7.50	8.10	7.50	7.50
Spray-dried bovine plasma	2.50	2.50	2.50	2.50	2.50
Fumaric acid^4^	0.46	0.46	0.46	---	---
Formic acid^5^	---	0.60	---	---	---
ZnO	---	---	---	---	0.40
Corn oil	2.00	2.00	2.00	2.00	2.00
Limestone	0.01	0.28	0.28	0.28	0.28
Monocalcium phosphate	0.75	0.75	0.95	0.75	0.75
Salt	0.40	0.40	0.78	0.40	0.40
L-Lys	0.55	0.55	0.55	0.55	0.55
DL-Met	0.24	0.24	0.24	0.24	0.24
L-Thr	0.24	0.24	0.24	0.24	0.24
L-Trp	0.08	0.08	0.09	0.08	0.08
L-Val	0.16	0.16	0.16	0.16	0.16
L-Ile	0.02	0.02	0.02	0.02	0.02
Vitamin premix^6^	0.25	0.25	0.25	0.25	0.25
Trace mineral premix^7^	0.15	0.15	0.15	0.15	0.15
Phytase^8^	0.06	0.06	0.06	0.06	0.06
Total	100	100	100	100	100
Calculated analysis					
SID amino acids, %					
Lys	1.35	1.35	1.35	1.35	1.35
Ile:Lys	52	52	53	52	52
Leu:Lys	112	111	113	112	112
Met:Lys	36	36	36	36	36
Met and Cys:Lys	56	56	56	57	57
Thr:Lys	64	64	64	64	64
Trp:Lys	21.1	21.1	20.8	21.1	21.1
Val:Lys	70	70	70	70	70
His:Lys	33	33	34	33	33
Total Lys, %	1.50	1.50	1.50	1.50	1.50
NE, kcal/kg	2,657	2,632	2,624	2,661	2,650
SID Lys:NE, g/Mcal	5.08	5.12	5.14	5.07	5.07
CP, %	19.4	19.3	19.5	19.4	19.4
Ca, %	0.36	0.46	0.46	0.36	0.36
P, %	0.51	0.51	0.51	0.51	0.51
STTD P, %	0.46	0.46	0.46	0.46	0.46
Ca:P	0.70	0.90	0.90	0.90	0.90
Na, %	0.40	0.40	0.40	0.40	0.40
Analyzed ABC-4, meq/kg	170	167	163	263	313

^1^Phase 1 diets were according to a feed budget of 2.3 kg/pig.

^2^Dairylac 80; International Ingredients Corporation; Fenton, MO.

^3^MEPRO; Prairie Aquatech; Brookings, SD.

^4^Primary Products Ingredients Americas LLC, Decatur, IL.

^5^Amasil NA; BASF; Florham Park, NJ.

^6^Provided per kg of diet: 4,134 IU vitamin A; 1,653 IU vitamin D; 44 IU vitamin E; 3 mg vitamin K; 0.03 mg vitamin B12; 50 mg niacin; 28 mg pantothenic acid; 8 mg riboflavin.

^7^Provided per kg of diet: 110 mg Zn from zinc sulfate; 110 mg Fe from iron sulfate; 33 mg Mn from manganese oxide; 17 mg Cu from copper sulfate; 0.30 mg I from calcium iodate; 0.30 mg Se from sodium selenite.

^8^Ronozyme HiPhos 2700 (DSM, Parsippany, NJ) provided an estimated release of 0.14% STTD P with 1,486 FYT/kg.

**Table 5.** Phase 2 diet composition (as-fed basis) Exp.2^1^

Fumaric acid	+	+	+	-	-
Low ABC-4 Strategy:	Low Ca	Formic acid	Crystalline lactose	-	-
ZnO:	-	-	-	-	+
ABC-4, meq/kg:	250	250	262	350	404
Ingredients, %					
Corn	57.61	56.74	58.53	57.80	57.55
Soybean meal	22.70	22.70	22.70	22.70	22.70
Whey permeate^2^	10.00	10.00	---	10.00	10.00
Crystalline lactose	---	---	8.00	---	---
Microbially enhanced soybean meal^3^	5.00	5.00	5.40	5.00	5.00
Fumaric acid^4^	0.46	0.46	0.46	---	---
Formic acid^5^	---	0.60	---	---	---
ZnO	---	---	---	---	0.25
Corn oil	1.00	1.00	1.00	1.00	1.00
Limestone	0.15	0.42	0.42	0.42	0.42
Monocalcium phosphate	0.85	0.85	1.00	0.85	0.85
Salt	0.55	0.55	0.80	0.55	0.55
L-Lys	0.53	0.53	0.53	0.53	0.53
DL-Met	0.23	0.23	0.23	0.23	0.23
L-Thr	0.24	0.24	0.24	0.24	0.24
L-Trp	0.08	0.08	0.09	0.08	0.08
L-Val	0.15	0.15	0.15	0.15	0.15
Vitamin premix^6^	0.25	0.25	0.25	0.25	0.25
Trace mineral premix^7^	0.15	0.15	0.15	0.15	0.15
Phytase^8^	0.06	0.06	0.06	0.06	0.06
Total	100	100	100	100	100
Calculated analysis					
SID amino acids, %					
Lys	1.35	1.35	1.35	1.35	1.35
Ile:Lys	55	55	55	55	55
Leu:Lys	113	113	114	113	113
Met:Lys	37	37	37	37	37
Met and Cys:Lys	56	56	56	56	56
Thr:Lys	64	64	64	64	64
Trp:Lys	21.9	21.8	21.9	21.9	21.9
Val:Lys	70	70	70	70	70
His:Lys	35	35	36	35	35
Total Lys, %	1.49	1.49	1.49	1.50	1.50
NE, kcal/kg	2,535	2,513	2,511	2,542	2,535
SID Lys:NE, g/Mcal	5.32	5.37	5.37	5.37	5.37
CP, %	20.5	20.5	20.6	20.5	20.5
Ca, %	0.44	0.55	0.55	0.55	0.55
P, %	0.55	0.55	0.55	0.55	0.55
STTD P, %	0.46	0.46	0.46	0.46	0.46
Ca:P	0.80	1.00	1.00	1.00	1.00
Na, %	0.35	0.35	0.35	0.35	0.35
Analyzed ABC-4, meq/kg	227	233	240	333	360

^1^Phase 2 diets were fed following phase 1 diets until d 24 post-weaning.

^2^Dairylac 80; International Ingredients Corporation; Fenton, MO.

^3^MEPRO; Prairie Aquatech; Brookings, SD.

^4^Primary Products Ingredients Americas LLC, Decatur, IL.

^5^Amasil NA; BASF; Florham Park, NJ.

^6^Provided per kg of diet: 4,134 IU vitamin A; 1,653 IU vitamin D; 44 IU vitamin E; 3 mg vitamin K; 0.03 mg vitamin B12; 50 mg niacin; 28 mg pantothenic acid; 8 mg riboflavin.

^7^Provided per kg of diet: 110 mg Zn from zinc sulfate; 110 mg Fe from iron sulfate; 33 mg Mn from manganese oxide; 17 mg Cu from copper sulfate; 0.30 mg I from calcium iodate; 0.30 mg Se from sodium selenite.

^8^Ronozyme HiPhos 2700 (DSM, Parsippany, NJ) provided an estimated release of 0.14% STTD P with 1,486 FYT/kg.

In both experiments, phase 1 and 2 diets were formulated to contain 1.35% standardized ileal digestible (SID) Lys. Following phase 2, all pigs were fed a common corn-soybean meal-based diet until the completion of the study on d 38. The common diet was formulated to 1.30% SID Lys and met or exceeded all other nutrient requirements established by the NRC (2012). Phase 1 diets were manufactured in pellet form while phase 2 and 3 diets were manufactured in meal form.

### Statistical Analysis

For both experiments, data were analyzed as a completely randomized design using the RStudio environment (Version 1.3.1093, RStudio, Inc., Boston, MA) of the R programming language [Version 4.0.2 (2020-06-22), R Core Team, R Foundation for Statistical Computing, Vienna, Austria] with pen as the experimental unit. Room/barn in each trial was included as a random effect. Differences between acidifiers used in Exp. 1 was tested by a contrast comparison of the four low ABC-4 diets with different acidifiers and excluded the diets not containing acidifiers. Similarly, the differences between strategies for Exp. 2 were tested by a contrast comparison of the three strategies to lower ABC-4 value of the diet and excluded the two high ABC-4 diets. In both experiments, the ABC-4 level effect was tested by a comparison of the mean of the low ABC-4 diets vs the single high ABC-4 not containing ZnO. The ZnO effect was tested by a pairwise comparison of the high ABC-4 levels with or without ZnO and excluded all low ABC-4 diets. Fecal DM were analyzed using the fixed effects of day, treatment, and the associated interaction accounting for repeated measures over time. Differences between treatments and day (where appropriate) as well as their interaction were considered significant at *P *≤ 0.05 and marginally significant at 0.05 < *P *≤ 0.10.

## RESULTS

### ABC-4 Analysis

All dietary analyzed ABC-4 values were less than calculated values regardless of treatment, phase, or experiment which is consistent with previous research (Lu et al., 2016; Stas et al., 2022). In Exp. 1, the analyzed ABC-4 values for diets without ZnO were on average 27 and 19 meq/kg lower than calculated values for phases 1 and 2, respectively. Analyzed ABC-4 values for the diet containing ZnO was 60 and 31 meq/kg lower than calculated values for phases 1 and 2, respectively. In Exp. 2, the analyzed ABC-4 values for diets without ZnO were on average 33 and 20 meq/kg lower than calculated values for phases 1 and 2, respectively. The analyzed ABC-4 value for the diet containing ZnO was 74 and 44 meq/kg lower than calculated value in phase 1 and 2, respectively. However, analyzed ABC-4 values still increased or decreased with respect to dietary formulation strategy and were similar among the low ABC-4 treatments within phase in each experiment.

### Experiment 1

There were no differences in growth performance between pigs fed the 4 dietary acidifiers for phase 1 (d 0 to 10), phase 2 (d 10 to 24), or the experimental period (d 0 to 24; Table 6). In the common period (d 24 to 38), there was a tendency observed (*P *= 0.094) with pigs previously fed KEM-GEST having numerically lower G:F. There were no differences in growth performance overall (d 0 to 38) or for any fecal DM collection period between pigs fed the 4 dietary acidifiers.

**Table 6.** Effects of acidifiers in diets formulated to equal ABC-4 on nursery pig performance and fecal dry matter (DM), Exp. 1^1^

Dietary acidifier:	1^2^	2^3^	3^4^	4^5^	-	-				
ZnO:	-	-	-	-	-	+				
Phase 1 ABC-4, meq/kg:	200	200	200	200	240	327		*P* =		
Phase 2 ABC-4, meq/kg:	250	250	250	250	290	344	SEM	Acidifier^6^	ABC-4^7^	ZnO^8^
Item: BW, kg										
d 0	6.1	6.1	6.1	6.1	6.0	6.0	0.02	0.970	0.795	0.953
d 10	7.3	7.2	7.3	7.4	7.3	7.6	0.14	0.796	0.726	0.254
d 24	11.3	11.2	11.2	11.1	11.1	11.8	0.28	0.934	0.752	0.072
d 38	20.7	20.4	20.2	20.2	20.3	20.6	0.57	0.946	0.907	0.683
Phase 1 (d 0 to 10)										
ADG, g	127	118	121	129	129	151	14.3	0.807	0.699	0.244
ADFI, g	143	135	141	146	151	160	13.3	0.773	0.395	0.518
G:F, g/kg	886	811	856	889	851	918	59.4	0.621	0.883	0.417
Phase 2 (d 10 to 24)										
ADG, g	283	282	281	266	268	296	18.3	0.626	0.495	0.146
ADFI, g	487	468	465	450	482	509	18.4	0.739	0.433	0.305
G:F, g/kg	582	603	603	587	552	582	32.1	0.669	0.009	0.135
Experimental period (d 0 to 24)										
ADG, g	218	213	214	209	210	234	11.6	0.939	0.781	0.131
ADFI, g	344	328	330	323	345	363	13.6	0.928	0.357	0.324
G:F, g/kg	635	643	648	645	607	646	25.0	0.973	0.020	0.052
Common period (d 24 to 38)										
ADG, g	672	657	646	654	662	628	43.2	0.848	0.810	0.107
ADFI, g	938	920	937	929	946	916	52.1	0.852	0.513	0.312
G:F, g/kg	716	715	690	705	699	684	11.1	0.094	0.376	0.199
Overall (d 0 to 38)										
ADG, g	383	376	373	373	376	379	15.5	0.980	0.990	0.880
ADFI, g	559	545	554	547	566	565	24.5	0.903	0.357	0.944
G:F, g/kg	685	689	675	683	664	668	7.0	0.310	0.013	0.652
Fecal DM, %^9^										
d 10	25.4	24.3	25.4	24.4	25.8	27.6	1.13	0.707	0.391	0.218
d 17	24.3	21.6	22.7	22.6	23.0	24.6		0.711	0.848	0.269
d 24	23.2	23.4	21.6	22.2	22.3	23.6		0.403	0.357	0.357

^1^A total of 300 pigs (initial BW of 6.1 ± 0.02 kg) were used in a 38-d nursery trial. A total of 6 dietary treatments were utilized with four low ABC-4 diets utilizing dietary acidifiers and two high ABC-4 diets with or without pharmacological levels on Zn from ZnO. Dietary acidifiers were added to achieve an equal ABC-4 level across the four low ABC-4 diets.

^2^Fumaric acid; Primary Products Ingredients Americas LLC, Decatur, IL. Included at 0.36% of the diet with an ABC-4 of -10,873 meq/kg.

^3^Activate DA; Novus, St. Charles, MO. Included at 0.87% of the diet with an ABC-4 of -4,487 meq/kg.

^4^KEM-GEST; Kemin, Des Moines, IA. Included at 1.01% of the diet with an ABC-4 of -3,800 meq/kg.

^5^ACID-AID; Alltech, Nicholasville, KY. Included at 0.84% of the diet with an ABC-4 of -4,680 meq/kg.

^6^Compares the four low ABC-4 diets. Excludes the high ABC-4 diets.

^7^Compares the mean of the low ABC-4 diets and the high ABC-4 without ZnO. Excludes the diet with ZnO.

^8^Compares the two high ABC-4 diets. Excludes all low ABC-4 diets.

^9^Treatment × day, *P* = 0.885; Treatment, *P *= 0.056; Day, *P *< 0.001. The *P*-values represented in the data table show the effect of treatment within day.

There were no performance differences between pigs fed either the low or high ABC-4 diets in phase 1 (d 0 to 10). In phase 2 (d 10 to 24) and the overall experimental diet period (d 0 to 24), pigs fed the low ABC-4 diets had increased (*P *≤ 0.020) G:F compared to pigs fed the 40 meq/kg higher ABC-4 diet. There were no differences between pigs previously fed either ABC-4 level for the common period (d 24 to 38). Overall (d 0 to 38), pigs fed the low ABC-4 levels had increased (*P *= 0.013) G:F compared to pigs fed the 40 meq/kg higher ABC-4 diet. There were no differences between pigs fed either ABC-4 level for all fecal DM collection periods.

There were no differences in growth performance between pigs fed diets with or without ZnO in phase 1 (d 0 to 10) or phase 2 (d 10 to 24). In the experimental period (d 0 to 24), pigs fed diets containing ZnO tended to have increased (*P *≤ 0.072) BW and G:F compared to pigs fed diets without ZnO. There were no differences between pigs fed diets with or without ZnO for the common period (d 24 to 38), overall (d 0 to 38), or for any fecal DM collection periods.

### Experiment 2

There were no differences in growth performance between pigs fed the three low ABC-4 formulation strategies for phase 1 (d 0 to 8; Table 7). In phase 2 (d 10 to 24) and the experimental period (d 0 to 24), pigs fed low Ca or the combination of acidifier strategies had increased (*P *< 0.05) ADFI compared to pigs fed the crystalline lactose diet. In the common period (d 24 to 38) and overall (d 0 to 38), there was a tendency observed (*P *≤ 0.094) with pigs fed the combination of acidifiers strategy having the greatest BW and ADG. Overall (d 0 to 38), pigs fed low Ca or the combination of acidifiers strategies had increased (*P *< 0.05) ADFI compared to pigs fed the crystalline lactose diet. On d 8, pigs fed the crystalline lactose diet had increased (*P *< 0.05) fecal DM compared to the combination of acidifiers strategy with the low Ca strategy intermediate.

**Table 7.** Effects of low ABC-4 formulation strategies on nursery pig performance and fecal dry matter (DM), Exp. 2^1^

Fumaric acid^2^:	+	+	+	-	-				
Low ABC-4 Strategy:	Low Ca^3^	Formic acid^4^	Crystalline lactose^5^	-	-				
ZnO:	-	-	-	-	+				
Phase 1 ABC-4, meq/kg:	200	200	193	300	387		*P* =		
Phase 2 ABC-4, meq/kg:	250	250	262	350	404	SEM	Strategy^6^	ABC-4^7^	ZnO^8^
Item: BW, kg									
d 0	5.9	5.9	5.9	5.9	5.9	0.09	0.175	0.497	0.758
d 8	6.7	6.7	6.6	6.6	6.8	0.07	0.436	0.099	< 0.001
d 24	12.5	12.7	12.4	12.4	13.2	0.29	0.132	0.268	< 0.001
d 38	20.5	21.0	20.3	20.3	21.0	0.30	0.068	0.394	0.073
Phase 1 (d 0 to 8)									
ADG, g	97	100	96	87	117	6.3	0.641	0.055	< 0.001
ADFI, g	120	117	113	114	134	5.7	0.492	0.611	< 0.001
G:F, g/kg	812	848	855	756	867	26.7	0.686	0.006	0.003
Phase 2 (d 8 to 24)									
ADG, g	368	377	361	362	397	15.7	0.138	0.407	< 0.001
ADFI, g	511	511	480	501	542	16.7	0.024	0.973	0.003
G:F, g/kg	722	738	753	724	734	28.5	0.165	0.126	0.345
Experimental period (d 0 to 24)									
ADG, g	277	284	273	270	303	9.7	0.162	0.200	< 0.001
ADFI, g	381	380	358	372	406	11.7	0.031	0.926	0.001
G:F, g/kg	731	750	763	728	748	24.6	0.202	0.022	0.054
Common period (d 24 to 38)									
ADG, g	566	589	565	568	556	16.7	0.094	0.625	0.405
ADFI, g	829	841	816	835	818	13.5	0.170	0.643	0.333
G:F, g/kg	684	702	694	680	681	14.9	0.649	0.062	0.928
Overall (d 0 to 38)									
ADG, g	384	397	381	379	396	7.9	0.079	0.288	0.068
ADFI, g	546	549	526	542	557	10.3	0.048	0.887	0.209
G:F, g/kg	704	722	724	700	711	6.5	0.789	0.005	0.128
Fecal DM, %^9^									
d 8	22.9	21.7	24.3	21.5	22.7	1.70	0.001	0.024	0.150
d 17	24.9	24.7	24.8	23.2	24.1		0.913	0.018	0.288
d 24	24.5	25.1	24.9	23.1	24.2		0.784	0.009	0.179

^1^A total of 725 pigs (initial BW of 5.9 ± 0.09 lb) were used in a 38-d nursery trial across three rooms. A total of 5 dietary treatments were utilized with three low ABC-4 diets utilizing different low ABC-4 formulation strategies. In addition, two high ABC-4 diets were utilized with or without pharmacological levels on Zn from ZnO.

^2^Primary Products Ingredients Americas LLC, Decatur, IL.

^3^Reducing the Ca:P ratio by 0.20.

^4^Amasil NA; BASF, Florham Park, NJ.

^5^Replacing whey permeate (Dairylac 80; International Ingredients Corporation, St. Charles, MO) with crystalline lactose based on lactose equivalence.

^6^Compares the 3 strategies to achieve a low ABC-4 level. Excludes high ABC-4 diets.

^7^Compares the mean of the low ABC-4 diets and the high ABC-4 diet without ZnO. Excludes the high ABC-4 diet with ZnO.

^8^Compares the two high ABC-4 diets. Excludes all low ABC-4 diets.

^9^Treatment × day, *P* = 0.414; Treatment, *P *< 0.001; Day, *P *< 0.001. The *P*-values represented in the data table show the effect of treatment within day.

In phase 1 (d 0 to 8), pigs fed the diets with low ABC-4 levels had increased (*P *= 0.006) G:F and tended to have increased (*P *≤ 0.099) BW and ADG compared to pigs fed the high ABC-4 diet. There were no differences between pigs fed either ABC-4 level for phase 2 (d 8 to 24). In the experimental period (d 0 to 24), pigs fed the low ABC-4 levels had increased (*P *= 0.022) G:F compared to pigs fed the high ABC-4 diet. In the common period (d 24 to 38), pigs previously fed low ABC-4 levels tended to have increased (*P *= 0.062) G:F compared to pigs fed the high ABC-4 diet. Overall (d 0 to 38), pigs fed the low ABC-4 level had increased (*P *= 0.005) G:F compared to pigs fed the high ABC-4 diet. For all fecal collection periods (d 8, 17, and 24), pigs fed the low ABC-4 levels had increased (*P *≤ 0.024) fecal DM compared to pigs fed the high ABC-4 diet.

For ZnO effects, during phase 1 (d 0 to 8), phase 2 (d 8 to 24), and the experimental period (d 0 to 24), pigs fed diets containing ZnO had increased (*P *≤ 0.003) BW, ADG, and ADFI compared to pigs fed the diet without ZnO. In addition, pigs fed diets containing ZnO had increased (*P *= 0.003) G:F in phase 1 (d 0 to 8) and tended to have increased (*P *= 0.054) G:F in the experimental period (d 0 to 24) compared to pigs fed the diet without ZnO. There were no differences between pigs previously fed diets with or without ZnO in the common period (d 24 to 38). Overall (d 0 to 38), pigs fed diets containing ZnO tended to have increased (*P *= 0.068) ADG compared to pigs fed diets without ZnO. There were no differences in fecal DM for pigs fed diets with or without ZnO for all collection periods.

## DISCUSSION

There has been extensive research regarding the use of dietary acidifiers as a method to improve growth performance and gastrointestinal health of weanling pigs. Generally, the use of dietary acidifiers has been proposed to decrease stomach pH which increases gastric pepsin activity, inhibits proliferation of pathogenic bacteria in the gastrointestinal tract, and improves nutrient digestion (Kil et al., 2011; Papatsiros and Billinis, 2012; Liu et al., 2018). Reduced stomach pH and increased gastric pepsin activity can be attributed to the acid-binding capacity characteristics of dietary acidifiers (Lawlor et al., 2005). Acidifiers typically have negative ABC-4 values which aids in reducing the ABC-4 of the complete diet. A negative ABC-4 value indicates the initial pH of acidifiers was below 4 and had to be titrated with sodium hydroxide to raise the pH (Stas et al., 2022). Although all acidifiers used in the current study were observed to have negative ABC-4 values, their individual ABC-4 varied between acidifiers which ultimately determined their inclusion in the diet. Fumaric acid has a 6,550 meq/kg lower ABC-4 value than the mean of the commercial acidifiers (Activate DA, KEM-GEST, and ACID-AID). The commercial acidifiers used in this study were all acid blends and included a carrier such as ground corn cobs. The use of a carrier dilutes the acid and increases the ABC-4 value of the final product. Therefore, in dietary ABC-4 formulation, a high inclusion of the commercial acidifiers is needed to achieve the same reduction in ABC-4 of the diet as a pure acid. Variation in ABC-4 between the three commercial acidifiers (Activate DA, KEM-GEST, and ACID-AID) is likely a result of different organic acids used and proportions of acids that make up each commercial acidifier.

In Exp. 1, a high inclusion rate of the three commercial acidifiers was used to get the same reduction in ABC-4 as the diet containing 0.36% fumaric acid because of the differences in their individual ingredient ABC-4. High inclusion of dietary acidifiers is often associated with poorer palatability of the diet, thus decreased ADFI. Walsh et al. (2007) and Ahmed et al. (2014) both observed pigs fed diets containing 0.4% of an acidifier blend decreased ADFI compared to diets without acidifiers. In contrast, in Exp. 1 we did not observe any significant differences in ADFI even though the three commercial acidifiers were included at more than double the rate of the previously mentioned studies. However, numerically the three commercial acidifiers decreased ADFI by 5.2% on average compared to the diet without acidifiers during the experimental period. In order to achieve the desired ABC-4 levels of 200 meq/kg from d 0 to 10 post-weaning and 250 meq/kg from d 10 to 24 post-weaning, acidifier blends may need to be included in the diet at an inclusion rate above supplier recommendations that could negatively impact feed intake. Therefore, other low ABC-4 formulation strategies should be incorporated along with dietary acidifiers to reach the desired ABC-4 level for weanling pigs without decreasing palatability.

In Exp. 2, three different low ABC-4 formulation strategies were used along with a dietary acidifier to achieve the target ABC-4 level. Fumaric acid was selected as the primary acidifier for Exp. 2 based off the results from Exp. 1. Previous research has also shown fumaric acid lowers the gastric pH of pigs when included in the diet (Bosi et al., 1999; Hanczakowska et al., 2011; Grecco et al., 2018). Among the three low ABC-4 formulation strategies, the combination of fumaric and formic acid had the greatest growth rate throughout the study and highest ending BW. This observation may be explained by possible synergistic effects of fumaric and formic acid that has been observed previously with other organic acids (Wei et al., 2021; Xu et al., 2022). However, pigs fed the low Ca and fumaric acid formulation strategy had similar performance to the combination of acidifiers during the experimental period. Previous research conducted by Warner et al. (2023) also observed benefits in weanling pig growth performance from feeding diets with low calcium carbonate levels and a dietary acidifier. The authors reported additive effects on growth performance from the two low ABC-4 formulation strategies which agrees with the results of the current study. While decreasing the amount of Ca in the diet is a method to decrease the ABC-4 level of the complete diet, another strategy could be using alternative Ca sources with low ABC-4 levels, such as calcium formate, to meet the desired Ca level (Lawlor et al., 2006). Lowering calcium carbonate in the diet to decrease ABC-4 without replacing it with an alternative Ca source would likely result in Ca levels below the pig’s dietary requirement. However, because this initial diet is fed for a short period of time, ramifications on bone growth would be expected to be minimal (Warner et al., 2023).

While the lactose replacement strategy did not affect ADG and G:F, using crystalline lactose to decrease the ABC-4 level of the diet resulted in a reduction in ADFI. A similar response was also observed by Nessmith et al. (1997) where pigs fed crystalline lactose had a 10.8% reduction in ADFI compared to pigs fed dried whey powder. The reduced ADFI could be a result of the palatability differences between the two lactose sources. Whey permeate contains easily digestible proteins and oligosaccharides that enhance the quality of the lactose source (Jang et al., 2021; Zhao et al., 2021). Therefore, pigs may have preferred diets containing whey permeate opposed to crystalline lactose because of their nutritional property differences. Although there was a decrease in ADFI, pigs fed crystalline lactose did have increased fecal DM on d 8. Reduced ADFI from pigs fed crystalline lactose may have resulted in less protein to be fermented in the gastrointestinal tract which could have increased the firmness of the feces (Diether and Willing, 2019). However, the main reduction in ADFI was observed when pigs transitioned to phase 2 diets and the increase in fecal DM from pigs fed crystalline lactose was observed on d 8 and not d 17 or 24. Although there were differences observed between the different low ABC-4 formulation strategies, on average, the low ABC-4 formulated diets improved feed efficiency and fecal DM compared to pigs fed higher ABC-4 diets.

In both experiments, the low ABC-4 diets improved feed efficiency compared to the high ABC-4 diets without ZnO. This observation agrees with previous studies formulated to the same low ABC-4 level of 200 and 250 meq/kg in phases 1 and 2, respectively ( Stas et al., 2025). The differences in ABC-4 levels between Exp. 1 and 2 explain the variable response to fecal DM. In Exp. 1, there was only a 40 meq/kg difference in ABC-4 levels with no effect on fecal DM. Whereas in Exp. 2, there was a 100 meq/kg difference in ABC-4 of experimental diets with low ABC-4 diets increasing fecal DM. There was also a discrepancy between both experiments for the response to ZnO. Significant improvements in growth performance for diets containing ZnO were only observed in Exp. 2, which is likely a result of the amount of variation between the two experiments. Pigs fed diets containing ZnO had a BW CV of 7.7 and 5.5% for Exp. 1 and 2, respectively. Even though variation was higher with Exp. 1, both experiments had approximately a 10.5% improvement in ADG when ZnO was added to the high ABC-4 diets, although significance was only able to be detected in Exp. 2. Overall, the addition of ZnO improved weanling pig performance as expected.

In summary, the results of both experiments indicate that many strategies and different dietary acidifiers can be used to achieve low ABC-4 diets to improve performance and fecal DM of weanling pigs in diets not containing ZnO. However, depending on the strategy to achieve a low ABC-4 target, the response in pigs may be affected. The use of crystalline lactose decreased feed intake but improved fecal DM shortly after weaning compared to low ABC-4 diets containing whey permeate. The combination of fumaric and formic acid had the greatest ending BW and ADG, but pigs fed diets with low Ca and fumaric acid was not statistically different. Fumaric acid had a much lower ABC-4 value and thus lower inclusion rates are required to achieve the same reduction in dietary ABC-4 as compared with commercial acidifiers. Commercial acidifiers can still be used to decrease the ABC-4 value of the diet to improve performance and fecal DM, but other strategies will likely need to be incorporated to obtain an ABC-4 level of 200 meq/kg from d 0 to 10 and 250 meq/kg from d 10 to 24 post-weaning. This study helps illustrate how different dietary acidifiers and formulation strategies can be incorporated into weanling pig diets to achieve an ideal ABC-4 level.
